# Steroid Recognition in Adhesion GPCRs: A Structural and Pharmacological Perspective

**DOI:** 10.1002/prp2.70221

**Published:** 2026-02-16

**Authors:** Bethany Fleming, Abdul‐Akim Guseinov, Irina G. Tikhonova, Graeme Milligan, Nicole A. Perry‐Hauser

**Affiliations:** ^1^ Centre for Translational Pharmacology, School of Molecular Biosciences University of Glasgow Glasgow UK Scotland; ^2^ School of Pharmacy Belfast Northern Ireland UK

## Abstract

Emerging evidence suggests steroids may act as generalizable ligands for adhesion GPCRs, a receptor class with considerable pharmacological potential. While most studies have focused on the ADGR‘G' subfamily, recent cryo‐EM structures of the androgens 5α‐dihydrotestosterone (5α‐DHT) and methenolone bound to ADGRD1 revealed a putative steroid‐binding pocket and enabled the rational design of a selective synthetic agonist. This perspective considers the broader significance of these findings amid some debate about the reproducibility and physiological relevance of steroid binding to adhesion GPCRs, reviewing all structural evidence and presenting comparative docking and homology modeling to evaluate conserved features and the plausibility of a shared recognition mechanism.

## 1 Introduction

Adhesion G protein‐coupled receptors (aGPCRs) are a structurally distinct class within the GPCR superfamily, characterized by long extracellular N‐terminal domains and a conserved GPCR‐autoproteolysis inducing (GAIN) domain. The GAIN domain facilitates receptor cleavage, exposing a tethered agonist (TA) sequence (the Stachel sequence) that enables self‐activation [1]. The seven‐transmembrane (7TM) domain of aGPCRs includes a large hydrophobic cavity which appears to have evolved to accommodate the hydrophobic TA consensus sequence [2, 3, 4, 5]. While this mechanism has advanced our understanding of aGPCR activation, it has not translated into pharmacological progress: the repertoire of known external agonists remains limited, and no approved drugs currently target this receptor class [6]. Given their involvement in diverse physiological and pathological roles, including immune regulation [7], neurodevelopment [8], and cancer [9], there is a pressing need to identify tractable ligands that could enable therapeutic intervention. A recent study published in *Cell* proposes that steroids may act as generalizable ligands for aGPCRs, offering a potential breakthrough in receptor modulation [10].

While several members of the ADGR‘G' subfamily have previously been shown to respond to steroid ligands [11, 12, 13, 14], the study by Yang et al. (2025) is the first to implicate steroid interactions beyond this group. Specifically, they demonstrated that the androgen 5α‐dihydrotestosterone (5α‐DHT), a potent agonist of the nuclear androgen receptor, can also activate the aGPCR ADGRD1 (also known as GPR133) in muscle cells, leading to elevated intracellular cyclic AMP levels and enhanced muscle strength [10]. This biological activity was supported by cryoelectron microscopy (cryo‐EM) structures of ADGRD1 in complex with 5α‐DHT, using two constructs that retained the 7TM bundle where 5α‐DHT is inferred to bind: one comprising the GAIN domain with a truncated N‐terminus and another representing a C‐terminal fragment lacking both the N‐terminal region and the Stachel sequence. These structures highlighted putative steroid‐recognition motifs conserved across multiple aGPCRs, suggesting the possibility of a generalizable steroid‐binding pocket. While these findings mark a significant advance in our understanding of steroid engagement for aGPCRs, it is noteworthy that many of the studies reporting steroid binding to different members of this family share authors in common and often involve collaborative efforts across laboratories. Such collaborations strengthen methodological rigor, but they also highlight the value of independent confirmation by fully separate research groups to establish the physiological relevance of these interactions. Recent evidence illustrates this point: a preprint examining ADGRG3 (also known as GPR97) reported limited functional activity for several previously proposed steroid ligands and argued against the physiological relevance of these interactions [15].

This perspective explores the broader implications of the 5α‐DHT/ADGRD1 interaction, particularly its potential to expand the druggable landscape of aGPCRs. To assess the generalizability of steroid recognition across this receptor family, we review available structural data on other steroid‐bound aGPCRs and perform comparative analysis using docking and homology modeling.

## 2 GPCR‐Mediated Non‐Genomic Actions of Steroids

In the classical genomic steroid signaling pathway, steroids bind cytoplasmic nuclear receptors, which then translocate to the nucleus to regulate gene transcription, producing physiological effects over minutes to hours. However, steroids can also elicit rapid, non‐genomic responses via membrane receptors. These include membrane‐associated forms of nuclear receptors (e.g., estrogen receptor ER**α**), as well as GPCRs [16, 17].

Several GPCR‐steroid interactions have been reported, though their acceptance varies. GPER1 is widely recognized as a functional estrogen receptor that mediates rapid signaling [18]. GPRC6A has been proposed to mediate non‐genomic androgen effects in vitro and in vivo, [19, 20, 21] but this claim remains contentious because of the receptor's broad ligand specificity and difficulties in reproducing results [22, 23]. GPR183 senses cholesterol metabolites (oxysterols) to direct cell migration in vitro and in vivo [24, 25], and this interaction has been corroborated by multiple groups, making it one of the more robust examples [26, 27]. In contrast, GPR17 was reported to respond to oxysterols [28], but these findings have not been confirmed by other laboratories and remain controversial due to conflicting data [29].

Together, these examples illustrate that, while GPCRs can act as steroid sensors, rigorous validation across laboratories is essential to distinguish genuine interactions from experimental artifacts.

## 3 Steroid Binding in the ADGR‘G' Subfamily

### 3.1 Glucocorticoid‐Bound ADGRG3

The first structural evidence of steroid binding to an aGPCR was reported in 2021 by Ping et al., who screened a panel of 23 endogenous steroid hormones and derivatives for activity at ADGRG3 [12]. They identified seven glucocorticoids, including cortisol and the exogenous ligand beclomethasone (BCM) [30], as activators of the receptor. Cryo‐EM structures of the receptor bound to BCM and cortisol revealed a long, ellipsoidal binding pocket within the seven‐transmembrane (7TM) domain, with a solvent‐accessible channel extending to the extracellular surface. The steroid cores were oriented perpendicular to the membrane and anchored by two hydrophobic patches and a polar contact at the base. The binding site included residues from TM1‐TM3, TM5‐TM7, and extracellular loops ECL2 and ECL3.

### 3.2 DHEA‐Bound ADGRG2

Lin et al. (2022) subsequently identified a group of adrenal‐derived steroids, including dehydroepiandrosterone (DHEA) and its sulfated form (DHEAS), as ligands for ADGRG2 (GPR64) [14]. Using a modified receptor construct to prevent autoproteolysis and stabilize G protein coupling, they first resolved the structure of apo‐ADGRG2 bound to Gs, revealing a two‐layered cavity within the 7TM domain: an upper region with potential to accommodate the Stachel sequence and a deeper pocket suggestive of ligand engagement. Screening 37 steroid hormones against this pocket, they identified four activators of ADGRG2—androstenedione, DHEA, DHEAS, and 20‐α‐hydroxycholesterol—and proceeded to characterize DHEA and DHEAS in detail, due to their higher efficacy.

Cryo‐EM structures of DHEA bound to both full‐length ADGRG2 and a truncated receptor lacking the N‐terminus and Stachel sequence revealed two distinct binding modes: an “upper” conformation involving a hydrogen bond with a structured water molecule, and a “lower” conformation deeper in the transmembrane region. The DHEA‐binding pocket shared partial structural similarity with the cortisol‐binding site in ADGRG3, particularly around ECL1 and TM2‐5, but diverged in ECL2, ECL3, and TM6‐7, resulting in an ~30° tilt of the steroid core. Despite these differences, both receptors featured three conserved hydrophobic residues (F^2.64^, W^6.53^, and F^7.42^) that directly interacted with their respective steroid ligands.

### 3.3 Progesterone Activates ADGRG6

Progesterone and 17‐hydroxyprogesterone (17‐OHP) were then identified as activators of ADGRG6 (also known as GPR126) in a steroid screen by An et al. (2022), both inducing Gi signaling [13]. While no structural data were resolved, key interaction residues were inferred through computational modeling, alanine scanning, and FlAsH‐BRET assays. Progesterone was modeled to lie flat and perpendicular to TM5, at ~60° relative to TM3, like cortisol in ADGRG3, while 17‐OHP adopted a perpendicular orientation to the membrane, representing a ~90° shift. Both ligands engaged overlapping hydrophobic and polar residues across TM1‐TM3, TM6‐TM7, and ECL2. Mutations in eight shared contact residues impaired receptor activation by both steroids, while selective mutations (e.g., K1001^ECL2^ and F1085^6.57^) disrupted responses to only one, underscoring distinct interaction patterns that could govern ligand‐specific recognition.

### 3.4 17α‐Hydroxypregnenolone Activates ADGRG1

The most recent steroid identified as an ADGRG family interactor was the DHEA prohormone 17α‐hydroxypregnenolone (17‐OH PREG), which was shown to activate ADGRG1 (also known as GPR56) and confer protection against ferroptosis‐mediated tissue injury [11]. Like the findings with progesterone, no direct structural evidence was provided for this interaction. Instead, the authors inferred the binding mode using a combination of computational modeling, alanine scanning, and FlAsH‐BRET assays. 17‐OH PREG was modeled in a perpendicular orientation to TM3, at ~60° relative to TM5, and engaged both hydrophobic and polar residues across TM3, TM5–TM7, and ECL2. Five residues contributing to the binding pocket—L^3.40^, W563 (ECL2), Y^5.36^, W^6.54^, and F^7.42^—were homologous to those involved in steroid binding in ADGRG3.

## 4 Structural Insights Into 5α‐DHT Binding to ADGRD1

### 4.1 5α‐DHT‐Bound ADGRD1

Although previous studies on steroid interaction with aGPCRs have focused on the ADGR‘G' subfamily, recent evidence suggests that this activity may extend more broadly. Yang et al. (2025) report that the androgen 5α‐dihydrotestosterone (5α‐DHT) activates ADGRD1, a member of the ADGR‘D' subfamily [10].

Androgens are known to elicit rapid, non‐genomic effects in muscle tissue and, as reviewed above, have been shown to activate GPCRs such as GPRC6A [17]. Notably, 5α‐DHT induces fast relaxant effects in bronchial smooth muscle, promotes uterine relaxation, and can trigger both contraction and relaxation in gastrointestinal smooth muscle. In the current study, 5α‐DHT was shown to enhance skeletal muscle contraction even in the presence of an androgen receptor antagonist, suggesting involvement of non‐classical receptors.

Through analysis of RNA sequencing data and subsequent validation experiments, the authors identified ADGRD1 as a candidate membrane receptor for 5α‐DHT that modulates muscle strength. To assess whether other steroid hormones could activate ADGRD1, a panel of 37 steroid hormones and their derivatives was screened. The results showed that only 5α‐DHT and its derivative methenolone (MET) modulated ADGRD1 activity, suggesting high specificity.

Cryo‐EM provided structural evidence of 5α‐DHT and MET binding to ADGRD1. Two receptor constructs were used: one containing the GAIN domain with a truncated N‐terminus (ADGRD1‐GAIN) and another comprising a C‐terminal fragment lacking both the N‐terminal region and the Stachel sequence (hereafter ΔTA‐ADGRD1‐CTF).

The ADGRD1‐GAIN complex with 5α‐DHT demonstrated two populations of the steroid‐bound state, with the steroid oriented either vertically (5α‐DHT^V^) or horizontally (5α‐DHT^H^) in a pocket spanning TM1‐TM3, TM5‐TM7, and ECL2. In contrast, ΔTA‐ADGRD1‐CTF adopted only the horizontal binding mode. Functional assays, mutagenesis studies, and binding free energy estimations suggested an energetic preference for the vertical steroid orientation, whereas 5α‐DHT^H^ represented a lower‐affinity interaction. Five residues specific to ADGRD1, but not conserved in other aGPCR members, were identified as critical for selective recognition of 5α‐DHT: Q^1.36^, L^2.64^, F^2.68^, F^3.40^, and W^6.53^.

MET, resolved in complex with ΔTA‐ADGRD1‐CTF, closely resembled 5α‐DHT^H^ but was oriented more parallel to the plasma membrane. The authors suggest that this difference is likely due to the presence of a double bond in MET that is absent in 5α‐DHT^H^.

### 4.2 Proposed Structural Motifs for Steroid Binding and Receptor Activation

Based on the binding mode of 5α‐DHT^V^ in the ADGRD1‐GAIN structure, Yang et al. proposed two key motifs involved in steroid recognition and activation of aGPCRs.

The first, termed the ΦFW motif, comprises residues Φ(F/L)^2.64^, F^3.40^, and W^6.53^, and mediates recognition of the hydrophobic tetra‐ring structure of the steroid core. W^6.53^ (W^6.48^ in class A GPCRs), a conserved and well‐characterized “toggle switch” residue, is often seen undergoing a conformational change upon agonist binding to promote receptor activation [31]; notably, 94% of aGPCRs retain a tryptophan at this position. In the 5α‐DHT^V^‐bound structure of ADGRD1, W^6.53^ directly contacts the steroid's A‐ring, with similar interactions involving either the A‐ring or D‐ring observed across other available aGPCR structures. The residues Φ(F/L)^2.64^ and F^3.40^ contribute to steroid binding through hydrophobic interactions or π‐π stacking with the steroid core.

The second motif, comprising residues F^7.42^ × ×N/D^7.46^, recognizes the polar functional groups of steroid hormones. In ADGRD1, F^7.42^ forms a π–π interaction with the double bond between the C3 and oxygen atoms of 5α‐DHT^V^, while N^7.46^ engages in a polar interaction with the carbonyl oxygen at C3. Similar interactions were observed in other steroid‐aGPCR structures.

The authors suggest that the high conservation of these motifs, present in over 70% of aGPCRs, supports a capacity for steroid recognition across the family.

## 5 Leveraging Steroid‐aGPCR Structures for Drug Discovery

### 5.1 AP503 is a Synthetic Agonist of ADGRD1

The possibility of a conserved steroid‐binding pocket across aGPCRs presents a compelling opportunity for therapeutic development. To this effect, Yang et al. used *in silico* screening to identify AP503 as a synthetic agonist of ADGRD1. AP503 was recently shown to alleviate osteoporosis in a mouse model, demonstrating the translational potential of targeting this pocket [32]. These findings demonstrate the feasibility of rational drug design for aGPCRs and support broader efforts to develop subtype‐selective modulators for diseases involving steroid‐responsive pathways. In the following section, we use comparative docking and homology modelling to assess how these structural models can inform computational drug discovery.

### 5.2 Steroid Specificity of the ADGRD1 5α‐DHT Binding Pocket

In the published ADGRD1 structure, 5α‐DHT was resolved in the binding pocket. Functional screening of 37 steroid‐like molecules revealed that only 5α‐DHT and its metabolite methenolone (MET) elicited receptor activation. To investigate why other candidate ligands remained inactive despite being screened at a high concentration (100 μM), we aligned all tested molecules based on the conserved C and D rings of the steroid backbone. This approach ensured that differences in binding were driven by substituent and ring modifications rather than the core scaffold.

**FIGURE 1:**
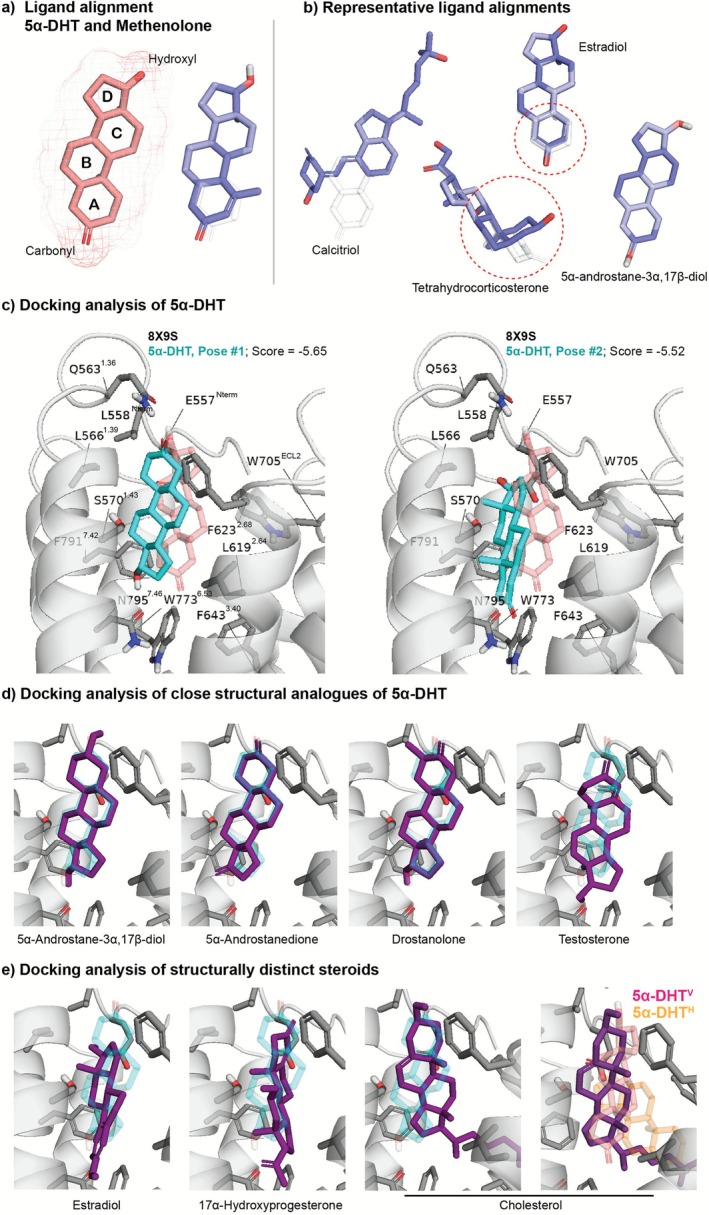
Structural alignment and docking of steroids to 5α‐DHT^V^. (a) Structure of 5α‐DHT, illustrating the conventional four‐ring steroid backbone (A‐D), with a carbonyl group on ring A and a hydroxyl group on ring D. Methenolone (right) differs from 5α‐DHT by a double bond in ring A. (b) Representative structural differences among steroids tested in Yang et al. study were aligned to 5α‐DHT to highlight key variations. Major differences include the polar substituents on rings A and D, and the presence/position of double bonds within the steroid nucleus. (c) Docking of 5α‐DHT to ADGRD1 (PDB: 8X9S), showing the two top‐scored poses (cyan) overlaid with the cryo‐EM ligand position (light red). Docking score is in kcal/mol. (d) Docking of close structural analogues of 5α‐DHT (dark purple), overlaid with the docked 5α‐DHT Pose #1. (e) Docking of structurally distinct steroids, including cholesterol, showing divergence in binding orientation relative to docked 5α‐DHT Pose #1 and cryo‐EM ligands. Docking was performed using the Schrodinger 2025–1 suite.

For the vitamin D derivatives calcitriol and calcifediol, along with related analogues, structural divergence from 5α‐DHT was pronounced. However, many other molecules differed more subtly (Figure 1A,B). Variations included altered scaffold stereochemistry (e.g., tetrahydrocorticosterone and 5β‐pregnane‐3,20‐dione), ring A aromaticity (e.g., estriol, estradiol, and estrone), and the presence of double bonds within the steroid nucleus, particularly at the junction of rings A/B or within ring A, as seen in MET. Additional modifications, such as methyl groups in ring A or hydroxyl groups in ring C, further altered steroid backbone geometry.

Two regions of consistent variability are located at the termini of rings A and D. While some modifications were substantial (e.g., at ring D in cholesterol and its analogues, ring A in stanozolol), most polar substituents were either hydrogen‐bond acceptor carbonyls or donor/acceptor hydroxyl groups.

Despite these observations, our structural analysis of the steroids failed to identify clear features that could account for the stark differences in ADGRD1 agonism between 5α‐DHT and its closely related analogues. For instance, 5α‐androstanedione differs from 5α‐DHT by a carbonyl substitution at ring D, while 5α‐androstane‐3α,17β‐diol differs by a hydroxyl substitution at ring A that disrupts a π–π interaction with F^7.42^, a mutation that only reduces 5α‐DHT potency tenfold (Figure 1B). Yet, neither ligand reportedly activates the receptor at concentrations up to 100 μM. Similarly, testosterone differs solely by a double bond in ring A and drostanolone by an additional methyl group in ring A, but neither compound elicits receptor activation. In contrast, MET, which carries a double bond and a methyl in ring A, does activate ADGRD1 with sub‐micromolar potency.

To gain further insight into steroid binding modes, we docked all tested steroid‐like compounds into the ADGRD1‐GAIN cryo‐EM structures with 5α‐DHT bound in “vertical” (PDB ID: 8X9S) orientation. Docking was performed using Schrodinger's GLIDE (Maestro, version 2025–1), with protein structures prepared via the default Protein Preparation workflow. It should be noted that re‐docking of 5α‐DHT to the ADGRD1 structure did not fully replicate the cryo‐EM pose, likely reflecting the inherent flexibility of steroid orientation within this pocket, a characteristic feature of symmetric, hydrophobic molecules. Nevertheless, comparative docking scores provide a useful metric for evaluating relative binding potential across receptors.

For 5α‐DHT, the top‐scoring docking poses generally resembled 5α‐DHT^V^, though there was notable structural diversity and only modest docking scores overall (−5.65 to −4.58 kcal/mol among the best 10 poses). The two highest‐ranked poses had similar scores: one aligned with the cryo‐EM orientation (−5.52 kcal/mol), while the other featured flipped A and D rings (−5.65 kcal/mol) Figure 1C. Distinguishing between these flipped‐ring poses based on cryo‐EM density alone is challenging, and molecular dynamics simulations may not resolve them due to high energy barriers. This potential conformational variability may complicate efforts to design selective modulators as structural changes in the ligand may differentially affect distinct binding modes, obscuring structure–activity relationships.

Across the steroid panel, docking results revealed a continuum of similarity in binding poses. Structurally similar ligands such as 5α‐androstane‐3α,17β‐diol, 5α‐androstenedione, and drostanolone docked to nearly identical binding modes to 5α‐DHT^V^, while testosterone showed higher deviation (Figure 1D). Others, including estradiol and 17α‐hydroxyprogesterone, adopted more divergent poses, often alternating between “horizontal” or “vertical” positioning. Some molecules, like cholesterol, occupied both sides of the pocket simultaneously (Figure 1E).

Taken together, these structural and docking analyses underscore the unpredictable impact of subtle steroid modifications on ADGRD1 engagement. This observation aligns with findings from a recent preprint by Bernadyn et al., which reported similar variability based on docking studies and the diverse orientations of steroids in cryo‐EM structures of steroid‐bound aGPCRs [15]. While such complexity may pose a significant challenge for *in silico* drug design targeting steroid‐responsive aGPCRs, the previous success of Yang and colleagues demonstrates that meaningful predictions are feasible, particularly when high‐quality structural data are available.

### 5.3 Pocket Similarity Across aGPCRs

To assess the potential of other aGPCRs to bind steroid ligands, we constructed homology models using the 5α‐DHT^V^‐bound ADGRD1 structure (PDB: 8X9S) as a template. Homology modeling was done in Schrodinger's Prime (Maestro, version 2025–1), with sequence alignments guided by the GPCRdb [33]. This approach yielded 21 models out of 28 attempted receptors, spanning all nine receptor subfamilies. These models assumed ADGRD1‐like position of corresponding residues, even when this disrupted continuous peptide loops, such as ECL2 or the N‐terminus. Their purpose was to illustrate the amino acid composition of the proposed steroid‐binding site, rather than to provide high‐confidence structural predictions, particularly in loop regions, which are poorly resolved at low sequence homology. Homology modeling was chosen over AlphaFold‐based predictions, which frequently mispositioned segments of the long N‐terminal peptide chain into the 5α‐DHT^V^ binding pocket, severely distorting relevant residue positions and conformations.

The most immediate observation from our structural analysis of the ADGRD1‐based models was the apparent interference from N‐terminal residues, which prevented a productive comparison of the steroid binding site across models. This interference could represent steric occlusion by residues from the N‐terminal loop or ECL2. However, given that our homology models often failed to predict accurate, or sometimes even realistic, loop conformations, such observations should be interpreted cautiously. Notably, several receptors with apparent clashes have experimentally resolved structures that do not support this occlusion. For example, ADGRG2 binds steroid ligands, yet its homology model suggests interference from N‐terminal sidechains that are unresolved in its cryo‐EM structure. Other receptors with modelled N‐terminal interference included ADGRA2 (GPR124), ADGRC1‐2 (CELSR1‐2), ADGRF5 (GPR116). Similarly, possible ECL2 clashes were observed in ADGRF5 (GPR116), ADGRG4 (GPR112), ADGRG6 (GPR126, which shows experimental steroid activation), and ADGRV1 (VLGR1), though loop modelling in these regions is inherently uncertain.

A more relevant finding is the presence of sequence diversity at positions likely to affect steroid binding. First, ADGRD2 (GPR144) and ADGRG7 (GPR128) showed notable sequence divergence from ADGRD1 at several aligned positions. For example, ADGRD2 has naturally occurring substitutions such as L619A, W705V, and N795Y, while ADGRG7 shows L619K, W705V, W773N, F791L, and N795I. This may reduce their compatibility with steroids like 5α‐DHT, although interaction with other steroid ligands cannot be ruled out.

**FIGURE 2:**
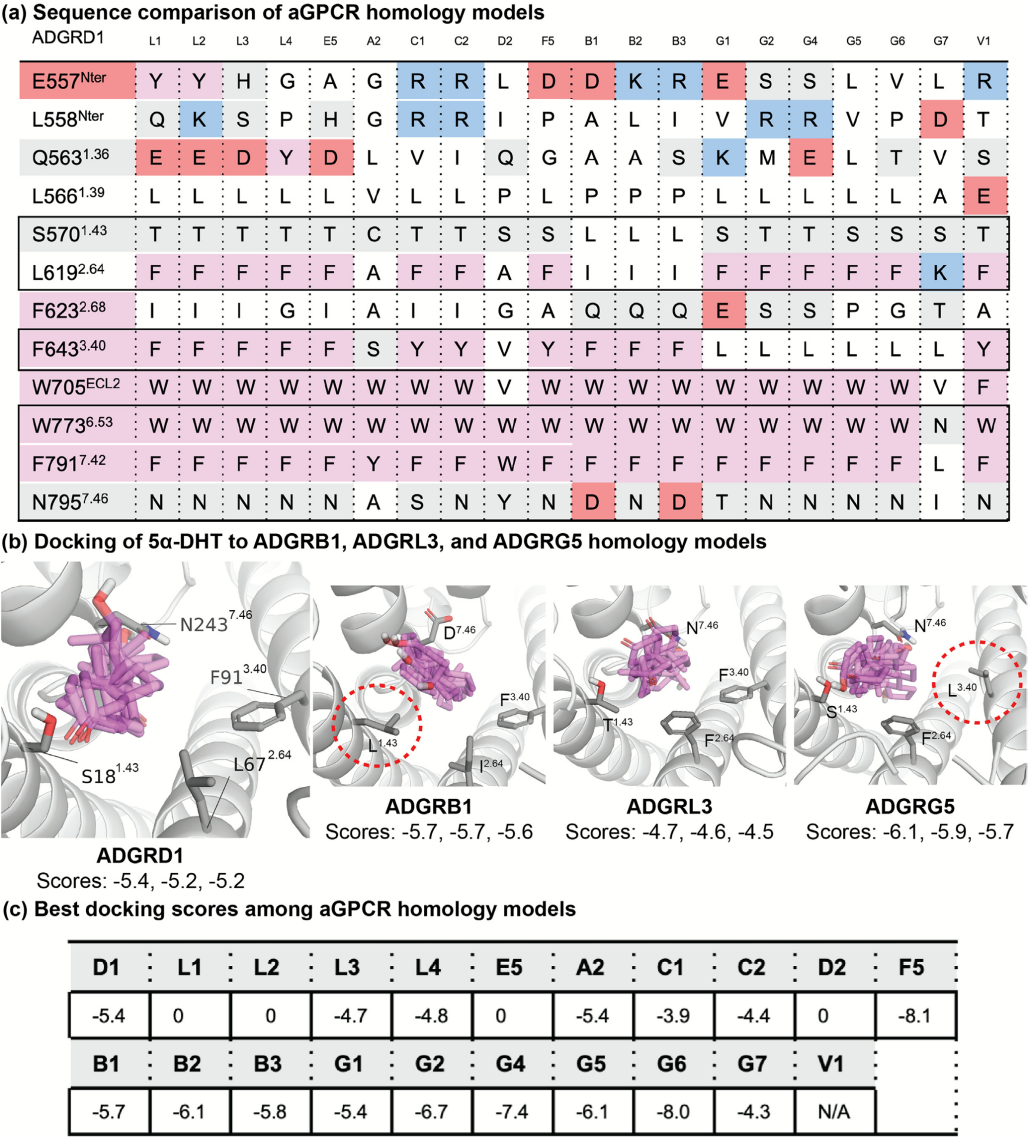
Homology model comparisons and docking results for selected aGPCRs. (a) Residue comparison near steroid‐binding site: Sequence alignment of residues within 5 Å of 5α‐DHT in the “vertical” ADGRD1 structure (PDB: 8X9S), across aGPCRs tested in Yang et al. and successfully modeled. Black brackets indicate residues located within the 7TM bundle. Residue types are color‐coded: Positive (blue), negative (red), polar (grey), and apolar (white). (b) Docking results: Top three docking poses for selected aGPCRs (ADGRL3, ADGRB1, and ADGRG5), with corresponding scores shown. (c) Docking summary: Best (lowest) docking score for each aGPCR model, indicated by subfamily letter and number (e.g., ADGRD1 as D1). A score of 0 indicates docking failure; “N/A” denotes failure to generate a docking grid. All docking scores are in kcal/mol.

In several receptors, only a few residues that interact with 5α‐DHT differ substantially from those in ADGRD1 (Figure 2A). For example, at position S^1.43^, ADGRB1‐3 (BAI1‐3) contain a leucine residue, introducing a bulky, apolar side chain. At position L^2.64^, multiple aGPCRs feature a phenylalanine substitution, and all ADGR‘G' members have a leucine at position F^3.40^. These substitutions are predicted to alter the shape of the steroid‐binding cavity, potentially impacting ligand accommodation. To evaluate their functional impact, we performed molecular docking of 5α‐DHT across the 21 aGPCR homology models, as previously described. However, due to the variability of docking poses, reliable predictions of steroid engagement and explanations for experimentally observed 5α‐DHT selectivity remain elusive. Nonetheless, our results suggest that substitutions at S^1.43^, L^2.64^ and F^3.40^ may significantly influence ligand binding.

When docked to receptors ADGRB1‐3 that have L^1.43^, 5α‐DHT repositioned further away from this residue compared to analogous docking to ADGRD1 (Figure 2B). Among aGPCRs with F^2.64^, docking to receptors with a bulkier F^3.40^ often failed entirely (Figure 2C), or produced only “horizontal” poses (e.g., ADGRF5) or poorly scored “vertical” poses (e.g., ADGRL3 and ADGRC1‐2) resembling those seen in ADGRD1. This suggests that, aside from differences in transmembrane helix packing, receptors with both F^2.64^ and F^3.40^ may be less compatible with steroid activation.

In contrast, when F^2.64^ was paired with a less bulky L^3.40^, docking yielded “vertical” poses that shifted toward L^3.40^ (Figure 2B,C), indicating a potential for steroid activation. These pose variations across aGPCRs with different residues at S^1.43^, L^2.64^, and F^3.40^ suggest that these positions may play a key role in determining receptor‐specific steroid selectivity.

## 6 Conflicting Evidence and Alternative Interpretations

### 6.1 Steroid‐Bound ADGRG3

A recent study reported discrepancies from the above steroid interactions with aGPCRs, specifically ADGRG3, challenging the notion of steroids as reliable agonists for these receptors [15]. Functional assays revealed that a mini‐panel of steroids, including previously proposed ADGRG3 ligands beclomethasone and cortisol, failed to elicit activity in gene expression assays or in an orthogonal GTPγS assay, whereas the known partial agonist 3‐α‐DOG retained mid‐micromolar potency on both ADGRG1 and ADGRG3 [34]. In neutrophil polarization assays, a TA peptidomimetic and 3‐α‐DOG induced robust polarization, while beclomethasone did not, a result further supported by agonist‐induced chemotaxis experiments. The authors also performed structural comparisons between corticosteroid‐bound ADGRG3 and TA‐bound aGPCRs. Unlike TA‐bound structures, which exhibit kinked TM6 and TM7 helices that facilitate G protein engagement and stabilize a fully active receptor conformation, corticosteroid‐bound ADGRG3 retained rigid helices and failed to stabilize a similar active‐like state. When analysing other steroid‐bound aGPCRs, the authors noted that steroid binding occurred in variable poses, like what we observed in our docking studies, contrasting with the highly conserved nature of the TA binding pocket. They argue that this variability may reflect nonspecific interactions driven by high concentrations of hydrophobic steroids, raising concerns about the physiological relevance of this interaction.

## 7 Conclusion

The recent cryo‐EM structures of 5α‐DHT and methenolone bound to ADGRD1 reveal a steroid‐binding pocket within the seven‐transmembrane domain that, when compared to previously resolved steroid‐aGPCR structures, supports the presence of two conserved motifs: Φ(F/L)^2.64^‐F^3.40^‐W^6.53^ and F^7.42^ × ×N/D^7.46^, which mediate hydrophobic and polar interactions with the steroid core, respectively. These features suggest a potential framework for steroid recognition that may extend to other aGPCRs. Importantly, these structural insights enabled the rational design of AP503, a synthetic agonist that selectively activates ADGRD1 without engaging other aGPCRs or the nuclear androgen receptor.

Taken together, these findings offer a compelling foundation for rational drug design targeting aGPCRs, with steroid scaffolds emerging as promising templates (Table 1). However, conflicting evidence, particularly from recent work revisiting steroid interactions with ADGRG3, underscores the need for corroboration across independent laboratories, especially given the history of inconsistent data in GPCR‐steroid studies. Detecting steroid interactions remains challenging due to their hydrophobicity, low potency, and potential for non‐specific effects in cell‐based assays. These same properties also complicate structural interpretation as steroids share amphipathic properties with detergents commonly used in membrane protein purification, raising the possibility that their presence in cryo‐EM structures may sometimes reflect non‐specific association rather than functional binding. Furthermore, in vivo evidence is limited; aside from the Yang et al. study on ADGRD1, no animal model has demonstrated disruption of steroid‐dependent physiological feedback loops following loss of an aGPCR.

**TABLE 1:** Adhesion GPCRs with published steroid interactions.

Receptor	Steroid	Structure (PDB)	Functional Evidence	PMID
ADGRG1	17‐hydroxypregnenolone (17‐OH PREG)		Activates ADGRG1‐G⍺12/13 fusion proteins in HEK239 cells; eliminates lipid peroxidation; attenuates liver injury	39 389 061
ADGRG2	Androstenedione (ASD)		cAMP accumulation in HEK293 cells; no G⍺q activation	35 982 227
	Dehydroepiandrosterone (DHEA)	7XKE, 7XKF, 7XKD	cAMP accumulation in HEK293 cells; no G⍺q activation	35 982 227
	Dehydroepiandrosteronesulfate (DHEAS)		cAMP accumulation in HEK293 cells; no G⍺q activation	35 982 227
	Deoxycorticosterone (DOC)		cAMP inhibition in HEK293 cells	35 982 227
	20‐⍺‐hydroxycholesterol		cAMP accumulation in HEK293 cells; no G⍺q activation	35 982 227
ADGRG3	Cortisol (Hydrocortisone)	7D77	cAMP inhibition in HEK293 cells	33 408 414
	Cortisone		cAMP inhibition in HEK293 cells and mouse Y‐1 cells	33 408 414
	Prednisolone		cAMP inhibition in HEK293 cells	33 408 414
	Prednisone		cAMP inhibition in HEK293 cells	33 408 414
	Dexamethasone		cAMP inhibition in HEK293 cells	33 408 414
	Beclomethasone (BCM)	7D76	G⍺qo3 Ca2+ mobilization in CHO cells; cAMP inhibition in HEK293 cells; no effect in neutrophil functional assays; negligible activation in GTPƔs assay	33 408 414; 22 575 658; 41 000 709
	11‐deoxycortisol		cAMP inhibition in HEK293 cells	33 408 414
ADGRG6	Progesterone		cAMP inhibition in HEK293 cells; no G⍺s, G⍺q, or β‐arr2 activation; promotes BC cell growth	35 394 864
	17‐hydroxyprogestrone (17‐OHP)		cAMP inhibition in HEK293 cells; weak cAMP accumulation; no G⍺q or β‐arr2 activation	35 394 864
	Testosterone		(weak cAMP accumulation)	35 394 864
	11‐deoxycortisol		(weak cAMP accumulation)	35 394 864
ADGRD1	5⍺‐DHT	8X9S, 9IV1	cAMP accumulation in HEK293 cells; G⍺s and G⍺12 activation; *Gpr133−/−* mice provide evidence for 5⍺‐DHT‐mediated modulation of muscle strength	39 884 271
	Methenolone (MET)	8X9U	cAMP accumulation in HEK293 cells; G⍺s activation	39 884 271

Finally, variability in steroid binding poses across resolved structures suggests that a universal mechanism for receptor–ligand engagement may be difficult to discern. Our own comparative docking and homology modelling did not reveal definitive conserved features, though these approaches have inherent limitations. Resolving these discrepancies will require systematic validation across receptor subtypes, experimental conditions, and independent research groups.

## Author Contributions

**Bethany Fleming:** writing – original draft, review and editing. **Abdul‐Akim Guseinov:** writing – original draft, review and editing; formal analysis (lead); investigation. **Irina G. Tikhonova:** software; writing – review and editing. **Graeme Milligan:** writing – review and editing; supervision. **Nicole A. Perry‐Hauser:** supervision, writing – original draft (lead), review and editing, visualization, conceptualization.

## Conflicts of Interest

The authors declare no conflicts of interest.

## Data Availability

The data that support the findings of this study are openly available in prp2025‐steroid‐adhesion‐gpcr‐dataset at https://github.com/perryhauserna/prp2025‐steroid‐adhesion‐gpcr‐dataset.
